# Sleep disorders and gout in Australian adults

**DOI:** 10.1186/s41927-021-00199-y

**Published:** 2021-08-28

**Authors:** Julia New-Tolley, Amy C. Reynolds, Sarah L. Appleton, Tiffany K. Gill, Susan Lester, Robert J. Adams, Catherine L. Hill

**Affiliations:** 1 Rheumatology Unit, The Queen Elizabeth Hospital, Woodville, SA Australia; 2 Flinders Health and Medical Research Institute (Sleep Health)/Adelaide Institute for Sleep Health, College of Medicine and Public Health, Flinders University, Bedford Park, SA Australia; 3 Adelaide Medical School, University of Adelaide, Adelaide, SA Australia

**Keywords:** Gout, Sleep Apnoea, obstructive, Sleep hygiene, Sleep disorders

## Abstract

**Background:**

The aims of our study were two-fold. Firstly, to determine if there is an association between gout and OSA in a representative Australian adult population. Secondly, to explore associations between gout and patient reported sleep outcomes.

**Methods:**

A cross-sectional national online survey of a representative sample of Australian adults > 18 years assessed self-reported doctor-diagnosed OSA, insomnia and patient reported sleep outcomes. Possible undiagnosed OSA was estimated using self-reported frequent loud snoring and witnessed apnoeas. Participants self-reported physician-diagnosed gout and other health conditions. Multivariable logistic regression analyses were performed for both objectives. Odds ratios with 95% confidence intervals were reported.

**Results:**

There were 1948 participants of whom 126 (6.5%) had gout and 124 (6.4%) had diagnosed sleep apnoea. After adjusting for age, body mass index (BMI), sex, alcohol intake and the presence of arthritis, those with obstructive sleep apnoea diagnosed on polysomnography were twice as likely to report having gout compared to those without. (OR = 2.6, 95% CI 1.5–4.6). Additionally, participants with symptoms suggestive of sleep apnoea were also twice as likely to have gout compared to those without (OR = 2.8, 95%CI 1.6–5.1). There was also a higher likelihood of restless legs syndrome, insomnia and worry about sleep in patients with gout.

**Conclusion:**

Diagnosed and suspected OSA are associated with higher likelihood of gout. Participants with gout are also more likely to report suffering from restless legs syndrome, insomnia and worry about their sleep. Given the morbidity associated with sleep problems, we should be vigilant regarding sleep health in our patients with gout.

## Introduction

Gout is the most prevalent inflammatory arthropathy with a prevalence of 6.8% in South Australia, and it is the most common form of inflammatory arthritis in men [1]. Gout is associated with co-morbidities including the metabolic syndrome and cardiovascular disease [2]. Obstructive sleep apnoea (OSA), which is also associated with cardiovascular morbidity, is also very common, with a self-reported prevalence of 8% in the Australian population [3]. A higher prevalence of incident OSA has been observed in older adults with gout [4]. A recent meta-analysis has shown an association between gout, serum uric acid and OSA [5]. Overall, it found that serum uric acid levels in patients with OSA were higher than in controls. In addition, individuals with OSA had a higher risk of developing gout, although this was not statistically significant (HR 1.25 95% 0.9–1.7).

The association between OSA and gout may be explained by intermittent airway obstruction during sleep leading to hypoxia, and resulting in increased serum uric acid via alteration in cellular metabolism [6]. Given the cardiovascular complications of both conditions, and treatability once diagnosed, this is an important area for further research.

There is likely to be a bidirectional relationship between sleep and gout, as gout flares may be influenced by circadian rhythms, and sleep quality is likely to be influenced by gout flares [7]. A study of 724 individuals with gout highlighted that attacks are twice as likely to occur at night [8]. We hypothesized that gout is therefore also likely to be associated with sleep disruption, which is known to lead to significant sequelae such as reduced work performance and road safety [3].

The aims of our study were two-fold. Firstly, to determine if there is an association between OSA and gout in a representative Australian adult population. Secondly, to explore associations between gout and patient reported sleep outcomes.

## Materials and methods

The aims of these study were addressed with a secondary analysis of the existing database of the Sleep Health Foundation 2019 web-based sleep health survey. The primary aim of this cross-sectional survey was to investigate prevalence of sleep disorders and sleep problems in the Australian population. The survey additionally included self-reported diagnoses of diverse chronic health conditions, including gout. Characteristics of the sample surveyed have been reported elsewhere [9]. Briefly, the sample incorporated Australian adults (> 18 years, *n* = 2044) recruited from an online survey panel by Dynata. The online panel comprises > 500,000 Australians, and allows for recruitment of a representative sample of Australian adults using a three-step randomisation process aimed at reducing recruitment bias [3, 9]. Participants were naïve to the content and aims of the survey during initial recruitment and screening to reduce bias. The CHERRIES checklist has previously been reported for this study, see Appleton et al. for details [9].

### Obstructive sleep apnoea (OSA)

Diagnosed OSA was determined by an affirmative response to the question *‘Have you ever been told by a doctor that you have any of the following conditions? Obstructive Sleep Apnoea’* (response items: yes, no, refused, don’t know)*.* Possible undiagnosed OSA (henceforth possible OSA) was determined based on the frequency in the past month of symptoms including observed frequent/loud snoring, and observed pauses in breathing/stopping breathing during sleep. Possible OSA was categorised as self-report of witnessed breathing pauses (i.e apnoeas).

Doctor diagnosed medical conditions were determined with the question *‘Have you ever been told by a doctor that you have any of the following conditions?...’* for gout, arthritis, high blood pressure, obesity, and heart disease. Response options were yes, no, refused or don’t know; participants were included in analyses if they provided either a yes or no response to the question on gout.

### Patient reported outcome measures for sleep

Patient reported outcome measures for sleep were assessed with the following questions and answer options included in Table 1.

**Table 1:** Patient reported outcomes for sleep

Outcome	Original question	Answer options. (Answers which coded for outcome are in italics)
Doctor diagnosed restless legs syndrome (RLS) or periodic leg movements during sleep (PLMS)	*Have you been diagnosed with restless legs or periodic leg movements of sleep*	1. *Yes* 2. No
Worried about getting to or maintaining sleep over 3 nights/week	*‘How often have each of the following things disturbed your sleep or kept you up at night in the past month?* - *Worry about getting to sleep or getting back to sleep after waking during the night’*.	1. rarely or never 2. a few nights a month 3. *a few nights a week* 4. *every or almost every night*
Discussing sleep with a health professional	During the last 12 months have you discussed your sleep with any of the following health professionals?’	(Multiple response) 1. *general practitioner* 2. *physiotherapist* 3. *chiropractor* 4. *specialist in private practice* 5. *hospital physician (with or without admission)* 6. *other physician* 7. *psychologist* 8. *psychiatrist* 9. *pharmacist* 10. *other (specify)* *Coded as*” *yes” if any one health professional indicated*
Pain which disturbs sleep	‘How often does pain stop you from going to sleep at night?’	1. Never 2. A few nights a week (1–3 nights/week) 3. *Most nights (4–6 nights/week)* 4. *Every night* 5. Don’t know
	*How often does pain wake you up at night?’*	1. Never 2. A few nights a week (1–3 nights/week) 3. *Most nights (4–6 nights/week)* 4. *Every night* 5. Don’t know
Adequate sleep	In the past month, how often have you experienced feeling you got adequate or satisfactory sleep’?	1. rarely or never, 2. a few nights a month, 3. *a few nights a week,* 4. *every/almost every night’*
Inadequate opportunity to sleep as a consequence of their typical routine	Does your current work schedule or typical weekday routine, including your duties at home, allow you to get enough sleep?	1. All/most of the time 2. *Sometimes* 3. *Rarely/never* 4. *Don’t know* 5. Refused

### Covariates

Age (years), sex (categories: male, female and other) and Body Mass Index from self-reported height and weight were included as covariates. Standard alcoholic drinks were self-reported, using the question: “*Thinking about alcoholic beverages such as beer, wine, liquor or mixed drinks, how many alcoholic beverages do you typically drink each week?”*. Responses were categorised to allow comparison to the Australian guidelines to reduce health risks from drinking alcohol [10] as *‘none’*, *‘<1 standard drink/day’*, *‘>1 to* *<2 standard drinks/day’*, and *‘>2 standard drinks/day’.*

### Data analysis

Data were analysed using IBM SPSS version 26.0 (IBM Corporation). Significant differences in sociodemographic and health characteristics of respondents by gout (yes or no) were examined using Mantel-Haenszel test of trend or Pearson χ^2^ statistic. Multivariable logistic regression analysis was used to examine associations between OSA (predictor) and gout (outcome) for Objective 1, adjusting for known correlates of gout (age, sex, BMI, alcohol consumption and arthritis).

**Fig. 1:**
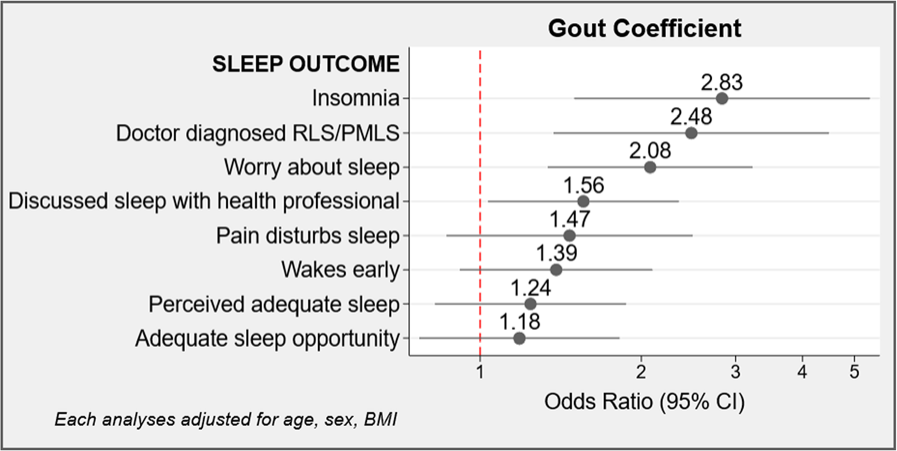
Coefficient plot reflecting odds of patient reported sleep problems by doctor-diagnosed gout from multivariable adjusted models

Multivariable logistic regression analyses were used to examine associations between gout (predictor) and each of the patient reported sleep outcomes (summarised in Fig. 1), with each model adjusted for age, sex and BMI, selected based on established relationships with sleep problems in community samples [3, 9]. Significance values for all models reported are based on the Wald statistic.

## Results

Of the 2044 respondents to the 2019 Sleep Health Foundation survey, 1948 (95.3%) provided a yes/no response when asked about gout. Respondents who indicated a diagnosis of gout (*n* = 126, 6.5%) were more likely to be male than female (11.2% v 2.0%, *p* < 0.001), and reported a higher prevalence of comorbidities including heart disease, arthritis, diabetes, high blood pressure and obesity than those without gout (see Table 2). Respondents with a diagnosis of gout were, on average, older than those who did not report a diagnosis of gout (M(+SD); 58.9(+ 16.6) years and (46.1 + 17.0) years respectively).

**Table 2:** Sociodemographic characteristics, risk factors and comorbidity and by doctor-diagnosed gout

	No Gout *(n = 1822)*	Gout *(n = 126)*	
	n (%)	n (%)	***p***^***a***^
**Sociodemographics**			
Sex			< 0.001
*Female*	981 (53.9)	20 (15.9)	
*Male*	839 (46.1)	106 (84.1)	
Age (years)			< 0.001
*18–24*	207 (11.4)	5 (4.0)	
*25–34*	346 (19.0)	10 (7.9)	
*35–44*	366 (20.1)	11 (8.7)	
*45–54*	312 (17.1)	14 (11.1)	
*55+*	591 (32.4)	86 (68.3)	
Location			< 0.001
*Metro*	1284 (70.5)	70 (55.6)	
*Rural/Regional*	538 (29.5)	56 (44.4)	
Current domestic status			0.87
*Never/Divorced/Separated/Widow*	749 (41.2)	51 (40.5)	
*Married/Partnered*	1069 (58.8)	75 (59.5)	
Income^b^			0.006
*<* *$30,000*	316 (19.8)	31 (25.6)	
*$30,001 - $50,000*	303 (18.9)	35 (28.9)	
*$50,001 - $100,000*	571 (35.7)	34 (28.1)	
*$100,001+*	410 (25.6)	21 (17.4)	
Financial strain			0.10
*Spend more than earn/get by*	689 (39.8)	59 (47.2)	
*Save a bit/save a lot*	1043 (60.2)	66 (52.8)	
**Risk factors**			
Body Mass Index (kg/m^2^)			< 0.001
*< 25*	663 (41.8)	25 (22.1)	
*25–29*	508 (32.0)	39 (34.5)	
*>* *30*	417 (26.3)	49 (43.4)	
Alcohol consumption			< 0.001
*None*	653 (35.8)	28 (22.2)	
*<* *1 standard drink/day*	476 (26.1)	26 (20.6)	
*1 -* *<* *2 standard drink/day*	110 (6.0)	14 (11.1)	
> 2 standard drink/day	583 (32.0)	58 (46.0)	
Smoking (current)	409 (22.7)	30 (23.8)	0.77
**Comorbidities**			
RLS/PLMS	121 (6.6)	23 (18.3)	< 0.001
Heart Disease	93 (5.1)	24 (19.7)	< 0.001
Arthritis	332 (18.3)	50 (41.0)	< 0.001
Diabetes	159 (8.8)	49 (39.8)	< 0.001
High Blood Pressure	409 (22.5)	82 (65.6)	< 0.001
Obesity	417 (26.3)	49 (43.4)	
**Obstructive sleep apnoea (OSA)**			
OSA			< 0.001
*No OSA*	1557 (85.5)	77 (61.1)	
*OSA symptoms*	169 (9.3)	21 (16.7)	
*OSA diagnosed with PSG*	96 (5.3)	28 (22.2)	

*Abbreviations*: *RLS/PLMS* Restless Legs Syndrome/Periodic Limb Movements in Sleep, *PSG* polysomnography

^a^significance values calculated from univariate χ^2^ analyses; ^b^ columns which do not add up to totals indicate missing data in this variable

### OSA and gout

The likelihood of reporting gout when a respondent also had OSA (no OSA, symptoms of OSA or diagnosed OSA with PSG) is reported in Table 2. Respondents who had OSA symptoms or diagnosed OSA were 2.6 and 2.8 times more likely respectively to report gout than those who did not have OSA. The model containing all variableswas statistically significant χ^2^(10, *N* = 1692) = 150.44, *p* < 0.001, explaining between 8.5% (Cox & Snell R^2^) and 22.1% (Nagelkerke R^2^) of the variance in gout, and correctly classifying 93.4% of cases (see Table 3).

**Table 3:** Multivariable logistic regression predicting likelihood of reporting gout by obstructive sleep apnoea status

	Wald	*df*	*p*	Odds Ratio	95% Confidence Interval	
					LLCI	ULCI
OSA^a^	18.93	2	< 0.001			
*OSA Symptoms*	11.49	1	0.001	2.81	1.55	5.11
*Diagnosed OSA*	10.65	1	0.001	2.61	1.47	4.64
Age (years)	13.20	1	< 0.001	1.03	1.01	1.04
Sex (male)^b^	24.95	1	< 0.001	5.25	2.74	10.07
BMI (kg/m2)^c^	4.91	2	0.09			
*25–29*	0.02	1	0.88	0.96	0.55	1.68
*≥* *30*	2.53	1	0.11	1.57	0.90	2.72
Alcohol consumption^d^	13.99	3	0.003			
*≤ 1 per day*	0.07	1	0.79	0.92	0.50	1.70
*1–2 per day*	3.38	1	0.07	2.00	0.96	4.18
*> 2 per day*	7.57	1	0.006	2.13	1.24	3.66
Arthritis^e^	4.86	1	0.027	1.65	1.06	2.58
Constant	148.60	1	< 0.001			

^a^ref no OSA; ^b^ref, female; ^c^ ref BMI < 25; ^d^ ref none; ^e^ ref no arthritis

### Gout and patient reported sleep problems

The results of multivariate adjusted logistic regression analyses for each sleep problem are presented in Fig. 1. Respondents with gout were 2.5 times more likely to report doctor diagnosed RLS/PLMS, were 2 times more likely to worry about their sleep, and were 1.6 times more likely to have discussed their sleep with a health professional than those without gout. Over two thirds (67.3%) of respondents with gout indicated that their routine allows sufficient sleep, and 62.8% felt that they regularly achieve adequate sleep. These findings were not significantly different to respondents without gout. In contrast, 15.0% of respondents with gout indicated they have doctor diagnosed RLS/PLMS compared to a prevalence of 6.7% in those without gout. Worry about sleep was also higher in respondents with gout (31.9% compared with 23.6%), and those with gout were more likely to have discussed sleep with a health professional than respondents without gout (38.9% compared with 28.5%).

## Discussion

In Australian adults, gout was more prevalent in those with diagnosed or probable sleep apnoea, after adjustment for relevant confounders. Of the 1948 participants, 126 (6.5%) had gout, and 124 (6.4%) had sleep apnoea diagnosed by polysomnography. A further 190 participants (9.8%) had symptoms suggestive of sleep apnoea without a formal diagnosis. Even after accounting for age, BMI, sex, alcohol intake and the presence of arthritis, diagnosis of gout was 2.8 times more likely in respondents with possible OSA. A participant with diagnosed OSA was 2.6 times more likely to have gout. Our results are in keeping with two large primary care matched retrospective cohort studies, in which patients with sleep apnoea were almost twice as likely to have gout [11, 12]. It is known than sleep apnea is under diagnosed in the community [13].

Our study was novel in that we found the association also emerged in those with suspected, but undiagnosed, sleep apnea. Our study design does not allow for consideration of the directionality of these relationships. However, a recent study by Singh and Cleveland showed a higher risk for OSA in patients with gout in the 5% United States Medicare beneficiary sample. They propose that two mechanisms are consistent between gout and OSA – specifically, inflammation and oxidative stress [4]. Prospective studies with gold-standard measurement of OSA, and inclusion of serum urate measures over time, will be required to further examine these relationships.

Our second aim was to explore the relationship between gout and patient reported sleep problems. Interestingly, participants with gout were twice as likely to have restless legs symptoms or periodic leg movements of sleep. Restless legs syndrome has been has reported associations with gout as well as many other comorbidities including obesity, diabetes, hypertension, thyroid disease and iron deficiency [14]. However, the mechanism behind the association between gout and restless legs syndrome is unclear. In fact, a 2019 study of 281 patients with restless legs syndrome showed reduced urate levels compared to controls matched by age and gender. It is worth noting however, that lower urate levels were also associated by increasing age, disease duration and haemoglobin level [15].

Given the prevalence of gout and the substantive financial burden of sleep-related conditions and flow on effects including productivity, employment, accidents and well-being, our findings highlight the importance of identifying and managing sleep problems in patients with gout [16]. Those with gout were also twice as likely to worry about sleep and 1.5 times as likely to have discussed sleep with a health professional. However, in those with gout, while there were trends towards issues with perceived adequacy of sleep and disruption of sleep secondary to pain, these effects were not significant. This result was surprising and not in keeping with our proposed hypothesis.

While gout is implicated in sleep related issues, this appear to be distinct from pain. One possible interpretation is that current pain may not be a primary contributor to sleep disruption in gout. Importantly, these findings highlight that simply reinforcing the need for regular, good quality sleep will not be sufficient in this patient group, as the majority have routines which allow for adequate sleep. Instead, it will be important to identify patient-specific sleep complaints and concerns and manage these on a case-by-case basis. Our findings are unique in linking gout with a diverse range of sleep outcomes beyond self reported duration and quality from a patient perspective, which provides important insight into the sleep health messaging and advice likely to benefit patients living with gout. Specifically, patients with gout differ from the general population, with reports of feeling they receive adequate opportunity to sleep. Given the relationship identified between OSA and gout, this patient group are more likely to benefit from messaging about assessment for sleep disorders rather than sleep hygiene (e.g. habitual routines, and allowing adequate opportunities to sleep).

One of the strengths of our study was the large sample that closely matched the general Australian population. It should be noted however, that while the sample was representative in regards to age, gender and geographical location across metropolitan and rural locations, there was a higher proportion of post-school qualifications, particularly Bachelor degrees or higher, than population estimates. We used self-reported doctor diagnosed gout, which has been shown to be reliable in epidemiologic studies [17]. Findings should be interpreted in the context of the study limitations. A key limitation is the cross-sectional nature of our study, so we were unable to comment on causation. An unmeasured shared risk factor for both gout and sleep apnea, is an important consideration, and a source of potential bias in our study. It is important to note that a large case control study using primary care data, published earlier this year, has shown that after adjustment for renal function, recent use of diuretics and heart failure, the association between gout and sleep apnea was no longer seen in males [18]. We were unable to correct for renal function as were limited to information collected via an existing patient questionnaire and did not have specifics related to treatments used.

Our study has highlighted that sleep disorders and gout are common and frequently comorbid in the Australian population. Sleep apnoea and gout are both associated with significant cardiovascular morbidity and mortality, but are also treatable. An awareness of the co-existence of both conditions should lead to increased screening and appropriate treatment tailored to patient needs. Further research is required to delineate the nature of the relationship between conditions, and also to establish if treatment of one condition may influence the trajectory of the other condition.

## Data Availability

The dataset(s) supporting the conclusions of this article is (are) included within the article (and its additional file(s)).

## Abbreviations

OSA: Obstructive sleep apnoea
BMI: Body mass index
RLS: Restless legs syndrome
PMLS: Periodic leg movements of sleep
